# Supplementary material for: Kinship networks of seed exchange shape spatial patterns of plant virus diversity
Source: Nat Commun. 2021 Jul 23;12:4505. doi: 10.1038/s41467-021-24720-6 (PMC8302746; doi:10.1038/s41467-021-24720-6)
Supplement: Supplementary file 1 — Supplementary Information New [file 41467_2021_24720_MOESM1_ESM.pdf]

Kinship networks of seed exchange shape spatial patterns of plant virus diversity

Marc Delêtre[1,*], Jean-Michel Lett[2], Ronan Sulpice[3], Charles Spillane[1]

[1] Genetics & Biotechnology Lab, Plant and AgriBiosciences Research Centre (PABC), Ryan Institute, National University of Ireland Galway, University Road, Galway H91 REW4, Ireland

[2] Centre de coopération Internationale en Recherche Agronomique pour le Développement (CIRAD), UMR PVBMT, Pôle de Protection des Plantes, 7 Chemin de l'IRAT, F-97410, Saint-Pierre, La Réunion, France

[3] Plant Systems Biology Lab, Plant and AgriBiosciences Research Centre (PABC), Ryan Institute, National University of Ireland Galway, University Road, Galway H91 REW4, Ireland

* To whom correspondence should be addressed: Marc Delêtre: deletrem@tcd.ie

Supplementary Information

Supplementary Methods

Ethnographic and Epidemiological Surveys. In addition to the eight villages surveyed between 2004 and 2007, four patrilineal communities were surveyed between 2014 and 2015 (Supplementary Data 1). Ethnographic surveys and data collection followed the same methodology as in Delêtre *et al.* (2011)[1] and was approved by the Research Ethics Committee of the National University of Ireland, Galway. In each community, we conducted 15-30 independent, semi-structured on-farm interviews. Farmers were asked to show all cassava landraces they grew and to specify when, where, how and/or from whom they acquired each landrace. Following their indications, leaf material was collected for genetic analyses (one sample $\times$ variety^{-1} $\times$ farmer^{-1}).

SSR Genotyping of Cassava Host Plants. Genetic diversity in cassava landrace populations from CCB and MVL was assessed using six nuclear SSR (simple sequence repeat) markers [GA12, GA21, GA57, GA126 (Chavarriaga-Aguirre *et al.* 1998)[2], and SSR55 and SSR68 (Mba *et al.* 2001)[3]]. PCRs were performed using Qiagen Multiplex PCR kits and phosphoramidite-labeled primers (MWG Biotech). All amplifications were carried out on 96-well PCR plates (Sarstedt AG & Co.) on a Biometra TProfessional 96-well gradient thermal cycler in 10 µL final volume. Amplification conditions followed the Qiagen protocol. Genotyping was performed on a 16-capillary ABIPrism 3130XL Genetic Analyzer (Applied Biosystems). Each genotyping plate was run along with six control samples from Delêtre *et al.* (2011)[1] to check for consistency across runs. Genotypes were extracted and analyzed using GENESCAN® Analysis 3.1.2 software (Applied Biosystems).

Population Genetic Structure of Cassava Landrace Populations. Population structure in cassava landrace populations was explored using the spatially explicit Bayesian clustering method implemented in BAPS (Corander *et al.* 2008)[4]. To avoid autocorrelation due to repeated multilocus genotypes (MLGs), only one copy of each unique MLG (n = 423) was used. Ten runs were performed for each value of K, the upper bound for the number of populations, ranging from 1 to 15. Admixture analyses were performed based on 100 iterations, with a minimum population size of 15 and 200 reference individuals from each population to calculate the admixture coefficient for individuals.

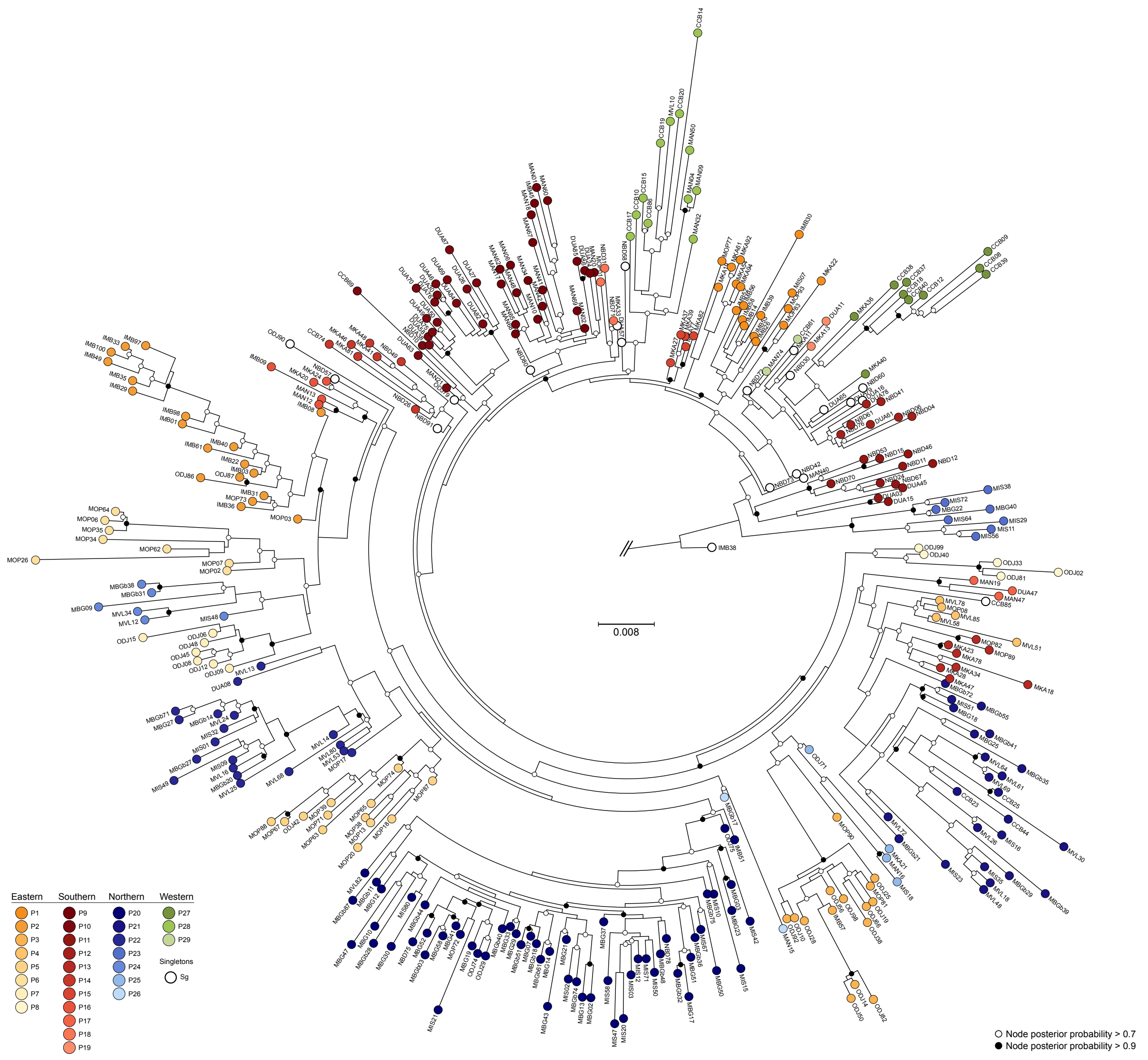

Supplementary Figure 1. Maximum-likelihood tree of 484 bp of the AC1 ORF coding for the replication-associated protein (Rep) from (*n* = 346) **ACMV isolates** collected in 2004, 2006-2007 and 2014-2015. Isolates are colored according to the 29 phylogenetic clusters (phylotypes) identified by Cluster Picker (Supplementary Data 2), based on the four regional clusters identified by BAPS (shades of blue for the northern cluster [MBG, MIS, MVL]), green for the western cluster [CCB], red for the southern cluster [DUA, MAN, MKA, NBD], and yellow for the eastern cluster [IMB, MOP, ODJ]). Singletons (haplotypes which were not assigned to any cluster) are shown as open circles. The tree was rooted using EACMV and EACMV-UG sequences as outgroups, including 12 EACMV-like sequences from Gabon.

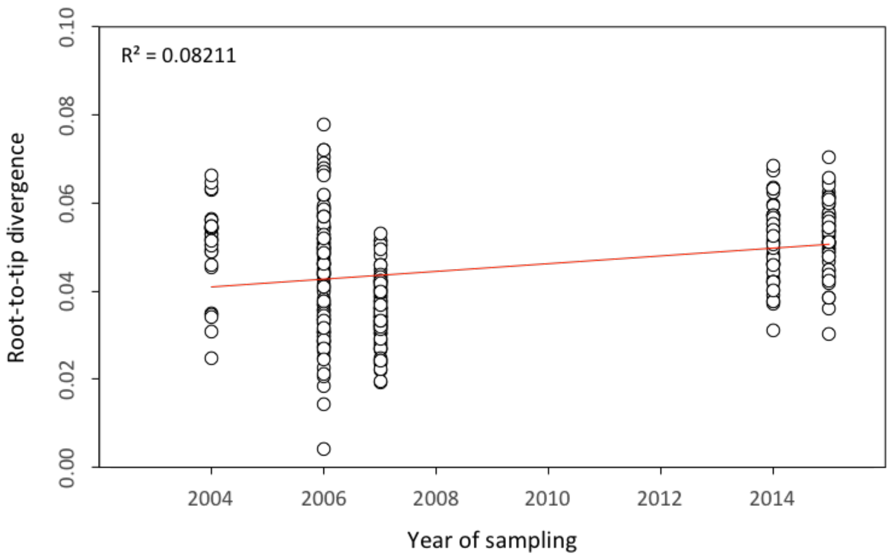

Supplementary Figure 2. Regression analysis of root-to-tip genetic distance against sampling time (year) using TempEst (Rambaut *et al.* 2016)[5]. The R^2 value reported is not a measure of the statistical significance of the regression but an informal measure of dispersion around the best-fit line and an indicator of the degree to which evolution has been clock-like (Drummond *et al.* 2003)[6]. The graph shows a lack of temporal signal despite the 9-year sampling gap between the 2006-2007 and 2014-2015 datasets.

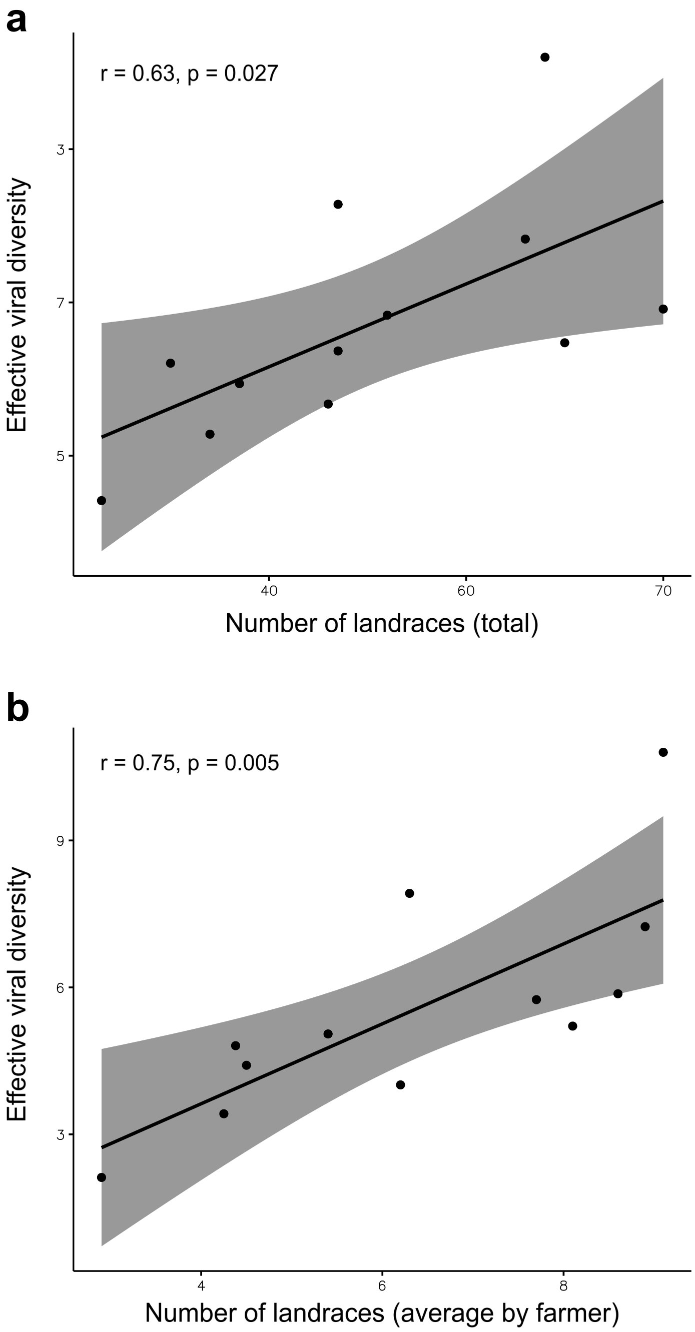

Supplementary Figure 3. Correlation between viral diversity (effective diversity based on Shannon diversity, ^{1}D) and varietal diversity (Pearson's correlation coefficient, *r*), expressed **a** as the total number of landraces recorded at the village level and **b** as the average number of landraces grown by farmers. Shaded areas represent 95% confidence intervals around the linear regression line. There was a positive correlation between viral diversity and varietal diversity (Pearson's correlation test, two-sided). Benjamini and Hochberg (1995)[7] FDR correction was applied to correct for multiple testing.

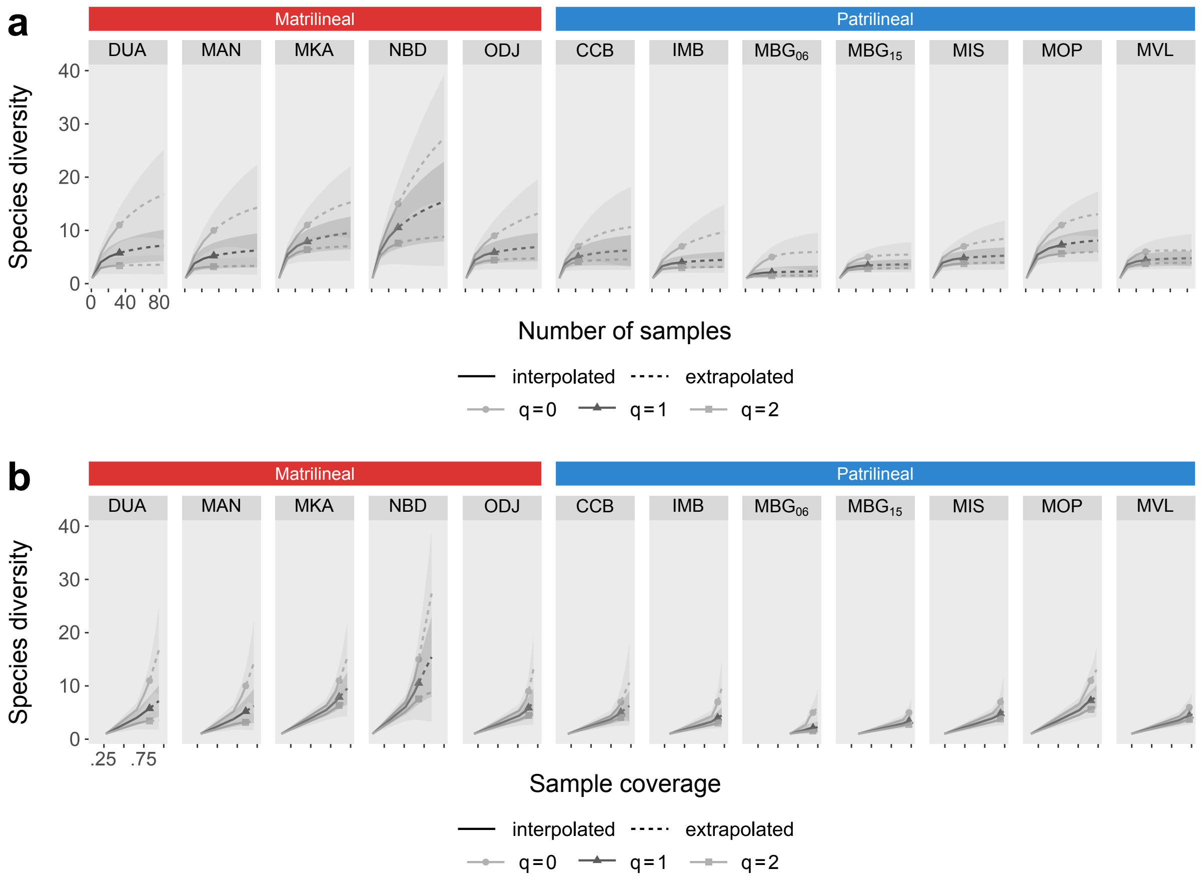

Supplementary Figure 4. Diversity profiles of viral populations in matrilineal and patrilineal villages. **a** Sample-size-based rarefaction curves (solid line) and extrapolation curves (dashed line) for phylogenetic diversity based on integral Hill numbers ^{q}D of orders $0 \leq q \leq 2$ (q = 0, species richness [circles]; q = 1, Shannon diversity; q = 2 [triangles], Simpson's diversity [squares]). **b** Coverage-based rarefaction curves and extrapolation curves. Extrapolation extends up to a base sample size of 85. Solid shapes represent reference samples. Shaded areas represent 95% confidence intervals.

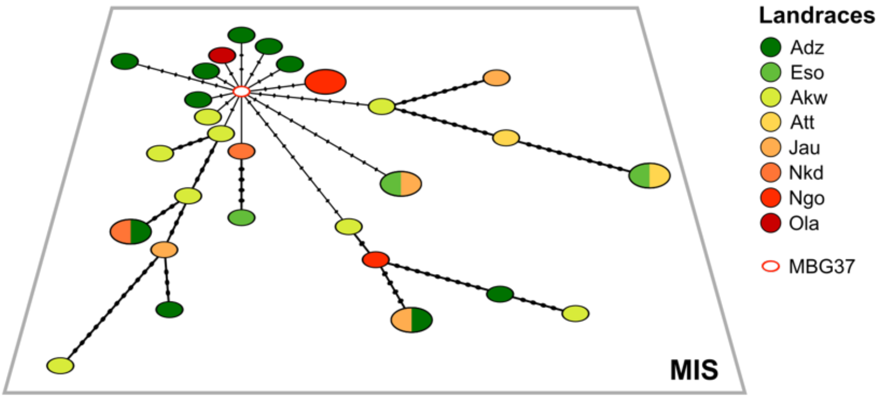

Supplementary Figure 5. Statistical parsimony network of ACMV genetic diversity in MIS. Each circle represents a distinct viral haplotype. Circle size is proportional to haplotype frequency. Genetic divergence is expressed as the number of mutational steps (black dots) between haplotypes. The varietal identity of the host plant is indicated with a different color and three-letter code. Although the haplotype was not sampled in the village, many viral strains sampled in MIS were closely related to MBG37 (circled in red).

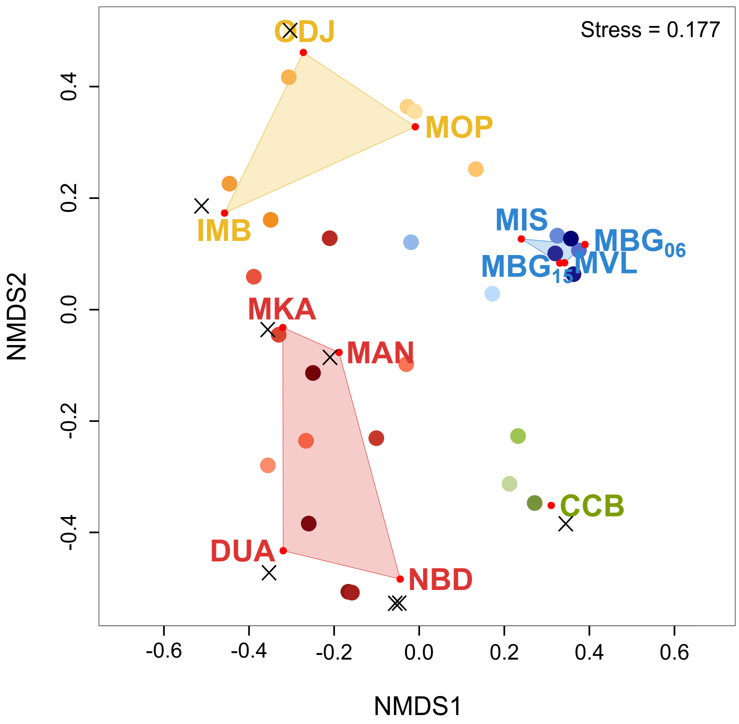

Supplementary Figure 6. Nonmetric multidimensional scaling (NMDS) analysis showing dissimilarity between viral assemblages based on their phylogenetic composition and Bray-Curtis distances. Dots represent the 29 phylogenetic clusters (phylotypes) identified by ClusterPicker (Supplementary Data 2). Colors are the same as in Supplementary Fig. 1. Crosses represent singletons. A permutational multivariate analysis of variance (PERMANOVA) (Anderson 2001)[8] showed that viral assemblages in patrilineal villages were significantly different in composition from viral assemblages in matrilineal villages (R = 0.153, P = 0.049).

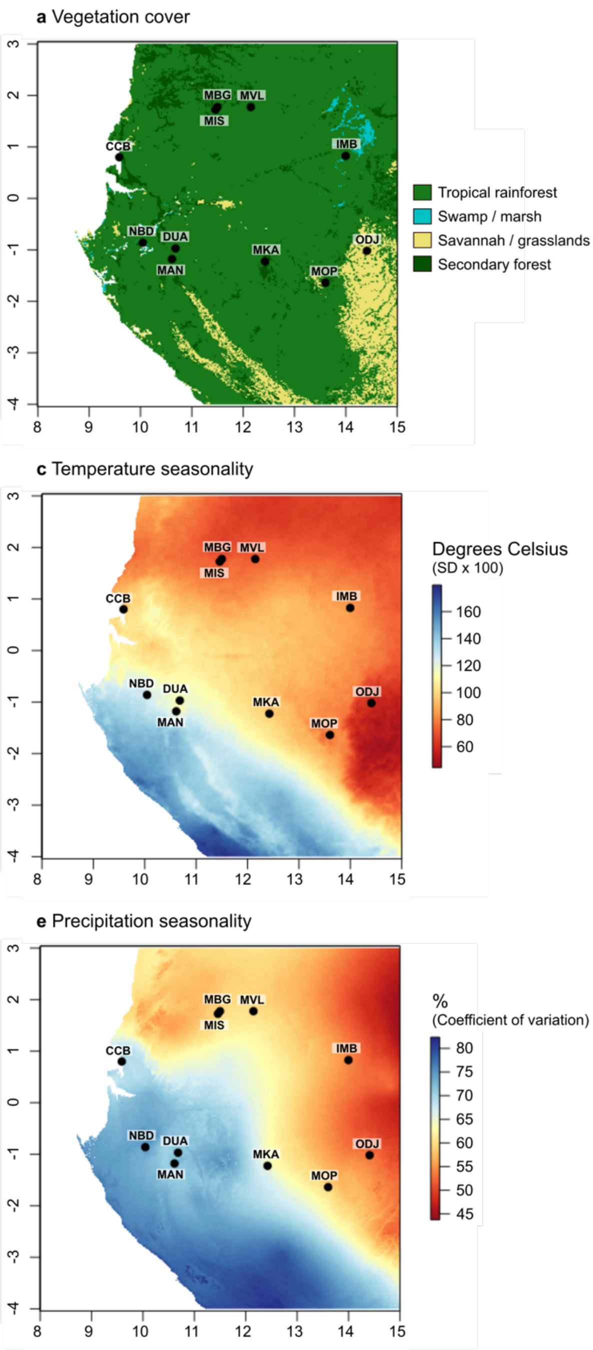

Supplementary Figure 7. Distribution of study sites in Gabon relative to several environmental parameters. **a** Raster data from the Global Land Cover 2000 Project (Mayaux *et al.* 2004)[9]. **b,c,d,e** Raster data from WorldClim 2.1 (Fick and Hijmans 2017)[10].

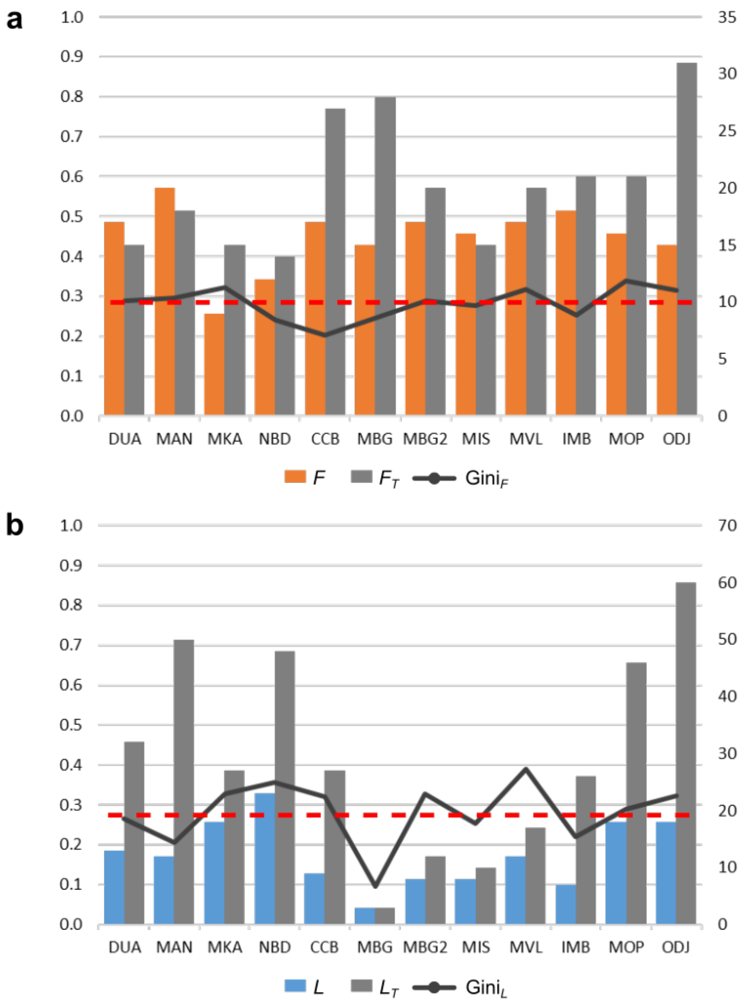

Supplementary Figure 8. Representativeness of datasets based on Gini coefficients relative to (a) farmers and (b) landraces. *F* (in orange) stands for the number of farmers and *L* (in blue) for the number of landraces included in the sample. F_T and L_T stand respectively for the total number of farmers interviewed and the total number of landraces recorded in each village. Gini coefficients measure inequality among values of a frequency distribution and range between 0 (complete equality) and 1 (maximal inequality). The average value across villages was 0.28 for both coefficients (dashed line). Sample representativeness with respect to households (**a**) was relatively balanced across villages, with the lowest inequality in CCB ($Gini_F$ = 0.202) and the highest in MVL (0.319) and MKA (0.324). In contrast, due to the high variation in varietal diversity at the village level, sample representativeness relative to landraces (**b**) was unbalanced across study sites, with near perfect equity for MBG ($Gini_L$ = 0.095) but a slight overrepresentation of one or several varieties in NBD (0.356) and MVL (0.390).

References

1. Delêtre, M., McKey, D. B. & Hodkinson, T. R. Marriage exchanges, seed exchanges, and the dynamics of manioc diversity. *Proc. Natl. Acad. Sci. USA* **108**, 18249–18254 (2011).
2. Chavarriaga-Aguirre, P. *et al.* Microsatellites in cassava (*Manihot esculenta* Crantz): discovery, inheritance and variability. *Theor. Appl. Genet.* **97**, 493–501 (1998).
3. Mba, R. E. C. *et al.* Simple sequence repeat (SSR) markers survey of the cassava (*Manihot esculenta* Crantz) genome: towards an SSR-based molecular genetic map of cassava. *Theor. Appl. Genet.* **102**, 21–31 (2001).
4. Corander, J., Sirén, J. & Arjas, E. Bayesian spatial modelling of genetic population structure. *Comput. Stat.* **23**, 111–129 (2008).
5. Rambaut, A., Lam, T. T., de Carvalho, L. M. & Pybus, O. G. Exploring the temporal structure of heterochronous sequences using TempEst. *Virus Evol.* **2**, vew007 (2016).
6. Drummond, A., Pybus O. G. & Rambaut, A. Inference of viral evolutionary rates from molecular sequences. *Adv. Parasit.* **54**, 331–358 (2003).
7. Benjamini, Y. & Hochberg, Y. Controlling the false discovery rate: a practical and powerful approach to multiple testing. *J. R. Stat. Soc. Series B Stat. Methodol*. **57**, 289–300 (1995).
8. Anderson, M.J. A new method for non-parametric multivariate analysis of variance. *Austral Ecol.* **26**, 32–46 (2001).
9. Mayaux, P., Bartholomé, E., Fritz, S. & Belward, A. A new land-cover map of Africa for the year 2000. *J. Biogeogr.* **31**, 861–877 (2004).
10. Fick, S.E. & Hijmans, R.J. WorldClim 2: new 1km spatial resolution climate surfaces for global land areas. *Int. J. Climatol.* **37**, 4302–4315 (2017).
