# Supplementary material for: Kinship networks of seed exchange shape spatial patterns of plant virus diversity
Source: Nat Commun. 2021 Jul 23;12:4505. doi: 10.1038/s41467-021-24720-6 (PMC8302746; doi:10.1038/s41467-021-24720-6)
Supplement: Supplementary file 6 — Reporting Summary [file 41467_2021_24720_MOESM6_ESM.pdf]

nature research

Corresponding author(s): Dr. Marc Delêtre

Last updated by author(s): Jun 5, 2021

Reporting Summary

Nature Research wishes to improve the reproducibility of the work that we publish. This form provides structure for consistency and transparency in reporting. For further information on Nature Research policies, see our Editorial Policies and the Editorial Policy Checklist.

Statistics

For all statistical analyses, confirm that the following items are present in the figure legend, table legend, main text, or Methods section.

n/a	Confirmed	
☐	☒	The exact sample size (n) for each experimental group/condition, given as a discrete number and unit of measurement
☒	☐	A statement on whether measurements were taken from distinct samples or whether the same sample was measured repeatedly
☐	☒	The statistical test(s) used AND whether they are one- or two-sided<br>*Only common tests should be described solely by name; describe more complex techniques in the Methods section.*
☒	☐	A description of all covariates tested
☐	☒	A description of any assumptions or corrections, such as tests of normality and adjustment for multiple comparisons
☐	☒	A full description of the statistical parameters including central tendency (e.g. means) or other basic estimates (e.g. regression coefficient) AND variation (e.g. standard deviation) or associated estimates of uncertainty (e.g. confidence intervals)
☐	☒	For null hypothesis testing, the test statistic (e.g. F, t, r) with confidence intervals, effect sizes, degrees of freedom and P value noted<br>*Give P values as exact values whenever suitable.*
☐	☒	For Bayesian analysis, information on the choice of priors and Markov chain Monte Carlo settings
☒	☐	For hierarchical and complex designs, identification of the appropriate level for tests and full reporting of outcomes
☐	☒	Estimates of effect sizes (e.g. Cohen's d, Pearson's r), indicating how they were calculated

Our web collection on statistics for biologists contains articles on many of the points above.

Software and code

Policy information about availability of computer code

Data collection	No software was used for data collection.
Data analysis	DNA sequence data was analyzed using CodonCode Aligner 7.0 (Codoncode Corporation, Dedham, MA, USA).<br>Analyses were performed using publicly available softwares: GENESCAN® Analysis 3.1.2 software (SSR genotyping), MUSCLE 3.8.31 (DNA sequence alignment), RDP4.101 (test for recombinations), FastTree 2.1.9 (phylogenetic analysis), TempEst 1.5 (testing for a temporal signal in the molecular phylogeny), Cluster Picker 1.3 (identification of phylotypes), BAPS 6.0 (Bayesian clustering analysis). All other statistical analyses were performed in R 3.5.1. The following packages were used: ineq (v.0.2-13), iNEXT (v.2.0.20), simboot (v.0.2-6), vegan (v.2.5-7), ecespa (v.1.1-13).

For manuscripts utilizing custom algorithms or software that are central to the research but not yet described in published literature, software must be made available to editors and reviewers. We strongly encourage code deposition in a community repository (e.g. GitHub). See the Nature Research guidelines for submitting code & software for further information.

Data

Policy information about availability of data

All manuscripts must include a data availability statement. This statement should provide the following information, where applicable:

- Accession codes, unique identifiers, or web links for publicly available datasets
- A list of figures that have associated raw data
- A description of any restrictions on data availability

All sequence data produced and analyzed in this study have been deposited in Genbank under accession numbers MT599318-MT599529 and MT599531-MT599676. Supplementary Information accompanies this paper: Supplementary Table 1 provides detailed information on the location and characteristics of the study sites. Supplementary Table 2 provides detailed passport data on the samples screened for cassava mosaic geminiviruses as well as Genbank accession

numbers and BLAST results for samples sent for DNA sequencing. Supplementary Table 3 provides ethnobotanical data from interviews with farmers. Supplementary Table 4 provides protocols for diagnostic PCR assays, with the names and sequences of primers used. Supplementary Fig.1 presents the phylogenetic tree built from ACMV sequence data. Supplementary Fig. 2 presents the regression analysis of genetic distance against sampling time. Supplementary Fig. 3 presents the correlation between viral diversity and cassava varietal diversity. Supplementary Fig. 4 presents diversity profiles for each community. Supplementary Fig. 5 presents the statistical parsimony network of ACMV diversity for MIS. Supplementary Fig. 6 presents the NMDS analysis of dissimilarity between viral assemblages. Supplementary Fig. 7 presents maps showing the location of study sites relative to different environmental parameters. Supplementary Fig. 8 shows the representativeness of datasets for each community based on Gini coefficients. Source data for figures 1, 2, 3 and supplementary figures S2 and S7 are also provided.

Field-specific reporting

Please select the one below that is the best fit for your research. If you are not sure, read the appropriate sections before making your selection.

☐ Life sciences ☐ Behavioural & social sciences ☒ Ecological, evolutionary & environmental sciences

For a reference copy of the document with all sections, see nature.com/documents/nr-reporting-summary-flat.pdf

Ecological, evolutionary & environmental sciences study design

All studies must disclose on these points even when the disclosure is negative.

Study description	This study investigates the role of social networks of seed exchanges between communities of smallholder farmers in the geographic spread of cassava mosaic geminiviruses (CMGs), the causal agents of the Cassava Mosaic Disease, a major threat to regional food security in Africa.
Research sample	The study focused on the African Cassava Mosaic Virus (ACMV) as a model. ACMV is ubiquitous in sub-Saharan Africa where cassava is cultivated. Unlike other CMG species, in which interspecific recombination and pseudo-recombination (the reassortment of heterologous genome components) is frequent, the ACMV genome presents also little evidence of recombination, making it easier to infer movements of viral lineages between communities of farmers using phylodynamic methods. Cassava (Manihot esculenta Crantz) plants were collected from 11 smallholder farming communities in Gabon (central Africa) between 2004 and 2007. In each village, 15-30 farmers were interviewed, their cassava farms visited, all cassava varieties were recorded and samples were collected for each variety during field visits in the presence of farmers. Depending on study sites, between 1 and 5 plants were collected for each variety and each farmer. Only one plant was later used for DNA analyses.<br>Total genomic DNA was extracted from dried cassava leaves. A total of 1132 plants (about 100 per village) were screened for the presence of CMGs, using diagnostic PCR. Plants infected by the African cassava mosaic virus (ACMV) only were sent for sequencing. 405 DNA sequences from ACMV representing ~484 bp of the replication-associated protein (Rep) open reading frame (ORF) AC1 were obtained.
Sampling strategy	In each study site, plants were selected haphazardly with at least one sample per variety per farmer in order to maximize the number of farmers and landraces comprised in the sample, taking care that no farmer or landrace was over-represented. Representativeness of local datasets was assessed by computing Gini coefficients relative to farmers and landraces.
Data collection	Marc Delêtre conducted on-farm surveys and collected the plant material in the field. He also carried out all labwork (DNA extractions and PCR amplifications) and data analyses. DNA sequencing of PCR amplicons was outsourced to Macrogen Inc. (Korea).
Timing and spatial scale	Data for ODJ were collected in 2004. Data for MBG, NBD, MOP, IMB were collected in 2006. Data for DUA, MAN, MKA were collected in 2007. Data for CCB and MVL were collected in 2014. Data for MBGb and MIS were collected in 2015.
Data exclusions	Data for two study sites, MYB and ODB, were excluded from epidemiological analyses at the onset of the study:<br>- MYB, which was surveyed in 2006, is a small neighborhood of hunter-gatherers (Bongo) which is part of Boumango, a large pluriethnic settlement (~3000 inhabitants) located ~45 km south of MOP and ~10 km from the border with the Republic of Congo. This village was excluded as it was not considered representative of cultural and local cassava varietal diversity.<br>- ODB was surveyed in 2007 and was also excluded because all local landraces in the village had been replaced in 2005 with planting material distributed by the local council in an effort to promote local agriculture, following a severe outbreak of anthracnose which resulted in a shortage of planting material. Molecular analyses of cassava plant material collected in ODB revealed clearly non-random patterns of diversity at the village scale as a direct consequences of this event (see Delêtre 2010 for details). Any effect of local dynamics of seed exchanges on viral diversity was canceled by the complete replacement of local germplasm with exogenous stem cuttings provided by the local council.
Reproducibility	Ethnobotanical surveys: Villages were chosen to represent contrasted situations in terms of social organization (patrilineal or matrilineal), ethnolinguistic diversity, size, accessibility and degrees of insertion with local/regional markets. In each community, a series of 15–30 independent, semistructured, on- farm interviews were conducted. Informants were randomly selected from among farmers willing to participate. Rarefaction curves were used to determine the minimum number of interviews required to ensure that the sample size was sufficient to record all cassava landraces present at the village level. Age, village of birth, and parental lineages were recorded for each farmer, and social networks describing farmer kinship relations were drawn. Maps of each village were also prepared, on which districts and the location of informants' houses were reported. Farmers were asked to name all cassava landraces they grow in their farms. Following their directions, up to five plants per landrace per farmer were collected.<br><br>Plant material collection: Leaf material was collected following farmers' directions. One to five plants per landrace per farmer were collected for genetic analyses.

	Molecular analysis: Genetic diversity in cassava landrace populations was assessed using six nuclear SSR (simple sequence repeat) markers. Each genotyping plate was run along with six control samples from previous runs (Delêtre 2010, Delêtre et al. 2011) to check for consistency across runs. To reduce the risk of typing errors, allele peaks were checked by eye. For detecting CMGs, samples were selected haphazardly in each community with at least one plant per variety per farmer in order to maximize the number of farmers and landraces comprised in the sample, taking care that no farmer or landrace was over-represented. PCR amplification was checked on 1% TBE agarose gel with positive and negative controls.
Randomization	No randomization was performed per se as samples used for the study were selected based on diagnostic PCR, targeting samples infected by ACMV only. See "sampling strategy" above for the selection of sampling material.
Blinding	No blinding was used during data collection. The patrilineal/matrilineal contrasted rules of seed transmission were an original, unexpected finding of the initial research carried out by the first author (Delêtre 2010), from which the majority of the data used in this study was drawn. Field surveys carried out in 2014-2015 targeted specifically patrilineal communities, in order to achieve a more balanced representation of patrilineal vs. matrilineal societies in the dataset and get a better coverage of northern Gabon. During fieldwork samples were collected solely on the basis of the varietal identity of the host plant, following farmers' indications and regardless of the plant's health status. All samples were identified by the name of the variety, a letter (corresponding to the farmer from whose fields the samples were collected), and a number (e.g., 'Adzoro A1'). A selection of samples (see above) were screened for infection by cassava mosaic geminiviruses. Those positive for single-infection by ACMV were selected for DNA sequencing and identified by a three-letter code corresponding to the study site and a number (e.g., 'IMB1').

Did the study involve field work? ☒ Yes ☐ No

Field work, collection and transport

Field conditions	All plant material was collected during visits of cassava fields in the presence of farmers and an interpreter. Except in 2004, 2014 and 2015, when fieldwork took place between March and June, fieldwork took place between September and December, during the transition between the dry and rainy seasons, a period corresponding to the time farmers weed and plant their new farms. Detailed information on local conditions (climate, elevation and agroecological factors) is provided for each study site in Supplementary Table S1.
Location	Detailed information on the location of study sites is provided in Supplementary Table S1.<br>CCB (01°00.408”N, 09°35.256”E, 15 m.a.s.l.)<br>DUA (01°02’41.4”S, 10°40’59.5”E, 37 m.a.s.l.)<br>IMB (01°02’19.3”N, 13°59’54.5”E, 527 m.a.s.l.)<br>MAN (01°17’17.2”S, 10°36’47.3”E, 52 m.a.s.l.)<br>MBG (02°08’17.9”N, 11°29’56.7”E, 600 m.a.s.l.)<br>MIS (02°04'43.7”N, 11°27'35.3”E, 600 m.a.s.l.)<br>MKA (01°20’24.8”S, 012°25’27.3”E, 314 m.a.s.l.)<br>MOP (01°49’09.1”S, 013°36’10.3”E, 485 m.a.s.l.)<br>MVL (02°09'26.8”N, 12°09.005”E, 600 m.a.s.l.)<br>NBD (00°55’08.3”S, 10°02’43.4”E, 20 m.a.s.l.)<br>ODJ (01°06’06.4”S, 14°24’37.9”E, 510 m.a.s.l.)
Access & import/export	The research project was hosted in Gabon by the Laboratoire Universitaire des Traditions Orales et Dynamiques Contemporaines (LUTO-DC) and the Université Omar Bongo (UOB), Libreville, who issued the research permits (N°0030/MESRIT/UOB/R, 76/MISPD/PHO/CAB, 00108/MENES/UOB/R, 00130/MESR/UOB/VRAAC, 00012/UOB/VRAAR, 00018/UOB/VRAAR).
Disturbance	No disturbances were caused during the study.

Reporting for specific materials, systems and methods

We require information from authors about some types of materials, experimental systems and methods used in many studies. Here, indicate whether each material, system or method listed is relevant to your study. If you are not sure if a list item applies to your research, read the appropriate section before selecting a response.

Materials & experimental systems

n/a	Involved in the study	
☒	☐	Antibodies
☒	☐	Eukaryotic cell lines
☒	☐	Palaeontology and archaeology
☒	☐	Animals and other organisms
☒	☐	Human research participants
☒	☐	Clinical data
☒	☐	Dual use research of concern

Methods

n/a	Involved in the study	
☒	☐	ChIP-seq
☒	☐	Flow cytometry
☒	☐	MRI-based neuroimaging
