# Supplementary material for: Kinship networks of seed exchange shape spatial patterns of plant virus diversity
Source: Nat Commun. 2021 Jul 23;12:4505. doi: 10.1038/s41467-021-24720-6 (PMC8302746; doi:10.1038/s41467-021-24720-6)
Supplement: Supplementary file 8 — Description of Additional Supplementary Files [file 41467_2021_24720_MOESM8_ESM.pdf]

Title: Supplementary Data 1.
Description: Typology of communities included in the CMD epidemiological survey in Gabon

Title: Supplementary Data 2.
Description: Passport and location data for host plant accessions and GENBANK accession numbers for corresponding viral DNA samples used in phylogenetic analyses

Title: Supplementary Data 3.
Description: Typology of farmers and origin of their cassava varieties

Title: Supplementary Data 4.
Description: Protocol for diagnostic PCR assays, with the names and sequences of primers used
